# A Qualitative Assessment of Community Acceptability and Use of a Locally Developed Children’s Book to Increase Shared Reading and Parent-Child Interactions in Rural Zambia

**DOI:** 10.5334/aogh.3920

**Published:** 2023-04-27

**Authors:** Jeanette L. Kaiser, Thandiwe Ngoma, Peter C. Rockers, Günther Fink, Allison Juntunen, Davidson H. Hamer, Ben Chirwa, Nancy A. Scott

**Affiliations:** 1 Department of Global Health, Boston University School of Public Health, Boston, MA, USA; 2 Department of Research, Right to Care Zambia, Lusaka, Zambia; 3 Swiss Tropical and Public Health Institute and University of Basel, Basel, Switzerland; 4 Department of Global Health, Boston University School of Public Health, USA; 5 Section of Infectious Diseases, Department of Medicine, Boston University School of Medicine, Boston, MA, USA; 6 Right to Care Zambia, Lusaka, Zambia

**Keywords:** emergent literacy, school readiness, early childhood care and education, early childhood development, maternal child health, sub-Saharan Africa, low- and middle-income countries

## Abstract

**Introduction:**

Early reading interventions hold promise for increasing language and literacy development in young children and improving caregiver-child interactions. To engage rural caregivers and young children in home reading, Zambian child psychologists and education specialists developed a culturally representative, local language children’s book targeted at pre-grade 1 children.

**Objectives:**

We qualitatively assessed community acceptability and use of the book distributed to households with young children in two provinces of Zambia.

**Methods:**

We conducted 15 focus group discussions (FGDs) with women (n=117) who received the “Zambian folktales adapted stories for young children” book. A codebook was created *a priori*, based on established themes in the guide; content analysis was conducted in Nvivo v12. Data were interpreted against the Theoretical Framework on Acceptability.

**Findings:**

Respondents described wide acceptability of the children’s book across multiple framework constructs. Respondents believed the book was culturally appropriate for its folktale structure and appreciated the morals and lessons provided by the stories. Respondents described using the book in multiple ways including reading in one-on-one or group settings, asking the child questions about the narrative or pictures, and providing additional commentary on the actions or figures in the pictures. Respondents believed the books were helping children grow their vocabulary and early literacy skills. The book’s simple vocabulary facilitated use by less educated caregivers. The primary concern voiced was the ability of low literacy caregivers to utilize the book for reading.

**Discussion:**

The children’s book was widely considered acceptable by rural Zambian communities. It provided a platform for an additional method of caregiver-child interactions in these households for reading, dialogue, and oral storytelling. Shared reading experiences have potentially substantial benefits for the language development and emergent literacy of young children. Programs to develop and deliver culturally acceptable books to households with limited access should be considered by governments and funders.

## Introduction

Early childhood care and education (ECCE) programs are increasingly accepted internationally as critical for ensuring children’s well-being. Program benefits are documented for academic achievement, labor market outcomes, health, and personal development [1,2]. ECCE programs focus on children aged zero to six, to ensure they have environments conducive to learning and cognitive development before entering formal education. However, in low- and middle-income countries (LMICs), many children lack access to ECCE programs, which contributes to an estimated 249 million children being at risk of not reaching their full developmental potential [1].

Emergent literacy is a particularly important aspect of child development [3]. Low literacy levels from early childhood can result in children falling behind their peers in early grades [4], with each passing year more challenging for them to catch up. These early disadvantages can lead to lower levels of educational attainment, reduced employment and income, and lower socio-economic status in adulthood [5]. However, caregiver-child interactions early in life, particularly verbal engagement such as reading aloud to children from birth, can assist development in language, vocabulary, cognition, and social emotional regulation, and eventually avert problems in literacy [6,7].

Throughout LMICs, at-home reading habits and availability of reading materials have shown a modest but consistent association with language development, social communication, emergent literacy, and broader school readiness scores [6,8,9,10,11]. Among a sample of over 100,000 children 36-69 months old analyzed across 35 LMICs using the multiple indicator cluster survey (MICS), the likelihood of being on track for literacy and numeracy was nearly double if at least one children’s book was in the household compared to households with no children’s books, after adjusting for confounders [9]. Yet, the presence of children’s books in LMIC households is sparse; only half of the children in the MICS sample had a children’s book at home [9]. Furthermore, interventions to encourage at-home reading have resulted in improvements in home reading practice scores with documented improvements in subsequent developmental outcomes in children [6,7,10,12].

Many programs worldwide seek to improve literacy among children and adults through providing age-appropriate books to households, schools, libraries, and community centers. Programs to distribute free books to children’s primary caregivers have been shown to improve children’s early learning environments [7,12]. In the United States, book distribution programs have been integrated into the pediatric primary care context for over 30 years. Since 1989, the Reach Out and Read program has been training pediatricians to counsel parents on the importance of reading to children and to hand out age-appropriate books at well-baby visits and has shown positive associations with improved vocabulary, reading activities and other developmental outcomes in young children [12,13]. Millions of books are distributed each year by small and large organizations, such as Book Aid International, Bibliotheques Sans Frontieres (Libraries Without Borders), the International Book Bank, Books for Africa, and The Dolly Parton Imagination Library, among countless others. Generally, these organizations seek to impact literacy among disadvantaged and vulnerable communities in low-, middle-, and high-income countries by overcoming one obstacle (lack of age-appropriate reading material). In fact, in a current attempt to close the learning gap from COVID-19 school closures in Zimbabwe, UNICEF distributed 200,000 culturally appropriate children’s storybooks to the most marginalized households [14].

Like other LMIC settings, literacy among primary school children in rural Zambia is low, and lack of pre-grade education disadvantages young children. Over the last few decades, the government of Zambia has made concerted efforts to ensure wider availability of ECCE programs in rural areas through community nursery/pre-primary programs; still, only approximately 30% of Zambian children have access to ECCE programs by age six [15,16]. Additionally, Zambian households have low-levels of child-appropriate reading materials at home. A recent study conducted in the capital city, Lusaka, found that only 22% of households with children in grade 1 owned a children’s book, and that ownership was predictive of early literacy skills [17]. Children’s book ownership is likely lower among rural Zambian households, which generally also have lower levels of income and parental years of education compared to urban households.

To engage rural Zambian parents and young children in at-home reading, a collaborative effort between Zambian researchers at the American Institutes for Research (AIR), The United States Agency for International Development (USAID) Zambia, and the START Foundation developed a book targeted at pre-grade 1 children to foster emergent literacy. The children’s book, designed to be culturally appropriate by a local Zambian team, was distributed in multiple rural districts by Right to Care Zambia. We, researchers from the Boston University School of Public Health and Right to Care Zambia, qualitatively assessed the community acceptability of the book among the female caregivers of young children and described how the book is being used within households and villages.

## Methods

### Study setting

This qualitative study was conducted in four rural Zambian districts: Choma, Pemba, and Kalomo districts of Southern Province and Nyimba district of Eastern Province, representing two of the ten provinces of Zambia. The populations of these districts are primarily rural, with limited access to electricity or improved sanitation [18]. In Zambia, schooling through grade 4 is in the local language of the province. English education courses begin in grade 2 with full-time instruction in English beginning in grade 5.

In Southern and Eastern Provinces, the majority (52% and 74%, respectively) of women aged 15 to 49 years have primary school education or less; only 8% and 4%, respectively, have completed secondary education [18]. These figures are similar for men aged 15-49 years; only 13% and 9% of men have completed secondary education in Southern and Eastern Provinces, respectively [18]. Sixteen to 50% of adult men and women cannot read at all in these provinces [18].

Low literacy levels are found among early grade learners as well. A large-scale assessment of grade 2 learners across rural Zambia in 2018 found that 64% “could not read a single word in the passage within a minute, indicating that the majority are non-readers (illiterate) [19].”

Rural Zambian districts also experience low utilization of ECCE programs. According to the 2018 Demographic and Health Survey, only 17% of five-year-olds in rural areas attended a pre-school program in 2018, and only 4% of caregivers indicated they read newspapers or articles at least once per week [18].

### Intervention description

The Zambian Folktales Children’s Book, titled “Zambian folktales adapted stories for young children,” was conceptualized by Mwaba Chipili, a Zambian psychologist, while working on an early childhood development (ECD) study in Southern Province. When adapting an ECD assessment tool for use with the rural Chitonga-speaking populations, Ms. Chipili reported being unable to find a children’s book with contextually relevant pictures and content, an important object used during the assessment [20]. Though not previously reported on in peer-reviewed literature, mothers participating in the ECD intervention, a learning group for parents of young children, identified lack of access to age-appropriate, non-school books for their children in a language they could understand as a barrier to their children reaching their full developmental potential [20] The importance of children’s literacy to parents and the scarcity of children’s books in rural Zambian communities has been corroborated by a study on traditional parenting practices in Zambia commissioned by the United Nations Children’s Fund (UNICEF) [21].

After not finding a children’s book with contextually relevant stories and pictures in Chitonga, Ms. Chipili sought funding and partners to fill this gap. The Zambian Folktales Children’s Book was funded by USAID through Global Health Program Cycle Improvement Project for artwork development and printing of the book, and AIR, for the translations and story validation workshops, described further below [20]. AIR has been working in partnership with the Zambian Ministry of General Education (MOGE) since 2001 assisting with information management, education planning, and assessment of learning outcomes [22].

To recognize the important oral folklore traditions within these communities and utilize that important source of caregiver-child interactions to foster emergent literacy, the multiple short stories that made up the book replicated the structure of folktales and storyline of common Zambian folktales [23]. Ms. Chipili led the storyboard development team of six individuals from the involved organizations [20]. Local Zambian folktales were compiled and selected for inclusion in the book by the storyboard development team based on story length, diversity of content, age appropriateness for children under five, and morals [20]. Some stories were derived from a dissertation on Bemba folktales housed in the libraries at the University of Zambia [23,24], while others were identified through community consultations, some occurring during a traditional parenting practices in Zambia study funded by the UNICEF [20,21]. Remaining stories were familiar to the storyboard development team from their childhoods, as told to them by their grandparents [20]. Selected stories were shortened, translated into Chitonga and five other Zambian languages, their vocabulary was simplified, and morals were clearly identified during preliminary adaptations [20]. Validation workshops were held with MOGE personnel and Dr. Jere-Folotiya, a Zambian educational psychologist with the University of Zambia, to ensure the final book was engaging and linguistically and conceptually appropriate for pre-grade children (i.e. before children enter grade 1 at age seven) [20]. The START Foundation designed the artwork for the stories [23].

Within a larger impact evaluation of an ECD intervention targeted at children under five years of age [25], Right to Care Zambia printed and distributed the Zambian Folktales Children’s Book in the catchment areas of fifteen rural health centers (12 in Southern Province and three in Eastern Province). Books were distributed through a variety of methods: house-to-house distribution by community-based volunteers (CBVs), by health facility staff and CBVs at health centers, or during community outreach activities. Books were printed in English, Chitonga, and Chinyanja. Households received books in their preferred language. Some large households received multiple books, sometimes in different languages if they chose. In total, 23,739 local language (Chitonga and Chinyanja) and English books were distributed to households between April 2020 and January 2022.

### Theoretical Framework

User or recipient acceptability is increasingly recognized as an important aspect of any development-focused intervention [26]. However, acceptability can be an unclear concept. Sekhon et al (2017) have offered a definition and theoretical framework for understanding intervention acceptability as “a multi-faceted construct that reflects the extent to which people delivering or receiving a healthcare intervention consider it to be appropriate, based on anticipated or experienced cognitive and emotional responses to the intervention [26].” Through their review of relevant literature, Sekhon et al (2017) developed a Theoretical Framework on Acceptability for healthcare interventions that divides the complex construct of acceptability into seven component constructs included with their definitions in Table 1 [26]. These component constructs look at how individuals feel about an intervention (affective attitude); how they perceive it fits within their value system (ethicality); their confidence in performing needed behaviors (self-efficacy); how well they understand the intervention and how it works (intervention coherence); their perception on its likelihood to achieve its purpose (perceived effectiveness); perceived effort required to participate in the intervention (burden); and what must be given up to participate in the intervention (opportunity costs) [26].

**Table 1:** Component constructs from the Theoretical Framework on Acceptability.


COMPONENT CONSTRUCTS	DEFINITION^a^	APPLICATION TO THE ZAMBIAN FOLKTALES CHILDREN’S BOOK INTERVENTION

Affective attitude	How an individual feels about the intervention	What community members feel about the book

Ethicality	The extent to which the intervention has good fit with an individual’s value system	How the book relates to the culture beliefs and value systems within these communities

Self-efficacy	The participant’s confidence that they can perform the behavior(s) required to participate in the intervention	How easy community members feel the book is to use What facilitates use of the book

Intervention coherence	The extent to which the participant understands the intervention and how it works	How the book is used within these communities

Perceived effectiveness	The extent to which the intervention is perceived as likely to achieve its purpose	What community members think about the effectiveness of the book

Burden	Perceived amount of effort that is required to participate in the intervention	What amount of effort community members think is needed to use the book
		What barriers or challenges exist to using the book

Opportunity costs	The extent to which benefits, profits, or values must be given up to engage in the intervention	What community members think must be given up to use the book


^a^ Definitions quoted from Sekhon M, Cartwright M, Francis JJ. Acceptability of healthcare interventions: An overview of reviews and development of a theoretical framework. *BMC Health Services Research* 2017; **17**. DOI: 10.1186/s12913-017-2031-8.

### Study design

We conducted 15 focus group discussions (FGDs) in the catchment areas of ten health facilities to understand community perceptions of the book, use of the book, and barriers and facilitators to use. Health facility sites were selected for qualitative data collection through stratified random sampling, ensuring each district was represented. The sites included four rural health centers from Kalomo district and two each from Pemba, Choma, and Nyimba districts.

FGDs were conducted with community women from households that had received a Zambian Folktales Children’s Book, had a child between three and nine years of age, and were resident in the selected catchment area. CBVs invited community members to participate in the FGDs, targeting individuals from a variety of villages within their respective areas.

### Data collection

The FGD guides included overarching questions for community perceptions of the children’s book and suggested probes and follow-ups to elicit additional detail. The guides were translated into the local languages of Chitonga and Chinyanja for use in the Southern and Eastern Provinces, respectively. The FGD guide is included as Supporting File 1.

A team of three data collectors were trained on research ethics, principles of qualitative data collection, and on the FGD guides during a three-day training in October 2020. Data collection lasted three weeks, from October to November 2020. Frequent handwashing with soap and water, use of alcohol-based hand sanitizers, wearing of face masks and social distancing were practiced during FGDs to protect participants and facilitators from transmission of SARS-CoV-2. FGDs were audio-recorded with consent from each participant.

All demographic information was collected using paper forms then later extracted using SurveyCTO® Collect software (V2.51; Dobility) on encrypted tablets. The paper forms were retained by project staff and filed in a locked cabinet for our records.

FGD respondents received a small token of appreciation for their time (e.g., a bar of soap and a small bottle of cooking oil) in accordance with the approved protocol and consent forms.

### Data analysis

Audio-recordings were concurrently translated and transcribed into Microsoft® Word. Boston University School of Public Health research staff (JLK & AJ) coded and analyzed the transcripts in NVivo v12 (QSR International, Doncaster, Australia). A mixed inductive-deductive approach was used for coding. An initial codebook for both the FGDs was created *a priori*, based on the established themes in the FGD guide. During the coding process, additional sub-nodes were added as themes emerged. A content analysis was conducted, analyzing the responses to each question asked in the FGD guide. The responses were then interpreted against the Theoretical Framework on Acceptability. Opportunity costs did not arise as a theme from the FGDs so respondent data could not be interpreted against the opportunity costs construct of the theoretical framework.

Demographic data were cleaned and analyzed in SAS v9.4 (Cary, NC). Means and standard deviations (SD) are presented for age, highest grade completed, and children in the household. Proportions are presented for respondent sex (female) and marital status.

### Ethics

Ethical approvals were obtained from the Boston University Medical Campus Institutional Review Board and the University of Zambia Biomedical Research Ethics Committee. Official government approval was granted by the National Health Research Authority, which is responsible for oversight of all research conducted in Zambia. Additional approvals were granted by the Ministry of Health at the national, provincial, and district levels. The overarching study was explained to the traditional chiefs overseeing the local areas, who all provided their endorsement.

Respondents were briefly screened for eligibility to participate in the FGDs. Written informed consent was obtained from each participant, documented with a signature or thumb print. When a thumbprint was required, an impartial witness (literate member of the community) was asked to observe the informed consent process and countersign that it occurred, and questions were answered. FGDs were conducted in local languages – Chitonga in Southern province and Chinyanja in Eastern province.

## Results

### Qualitative respondent characteristics

Qualitative respondents (n = 117) were all female and primary caregivers to children between three and nine years of age. They were generally married or cohabiting (81.2%), on average 33.1 years old (SD 10.4 years), with 7.5 years of education (SD 2.8 years), and had 2.8 children (SD 1.2) under 10 years of age living in their households.

### Acceptability component construct: Affective attitude

Generally, respondents voiced very favorable views of the children’s books and perceived recipient households as appreciative and happy to have received them (Table 2). They reported that children enjoyed the books and asked parents to read to them. Respondents particularly liked the folktale nature of the stories, which were perceived to connect readers with their culture and ancestry and engaged individuals of all ages. They stated that elders in the community looked fondly on these stories and found value in sharing the folktales of older generations as they could reconnect with the ways they learned as children. While the book was targeted at younger children, respondents frequently stated that the stories and lessons were appropriate, educational, and entertaining for all ages.

**Table 2:** Community perceptions of Zambian Folktales Children’s Book content and use by theoretical framework construct, including illustrative quotes.


COMPONENT CONSTRUCT	THEMES	ILLUSTRATIVE QUOTES

**Affective Attitude**	**1. Positive views from caregivers** **2. Engages & entertains children:** children enjoy the book and are happy to read; ask parents to read to them **3. Appreciate folktales stories:** help connect with ancestry; their grandparents were taught some stories as children	“I heard that the majority of people are praising this book because the majority of children are kept busy with this book. Even those who do not know how to read can point at pictures, here is a cow, this is a goat.” – Community member, female, Pemba district “It made me happy that this book teaches everything that is in the country Zambia. It teaches you good and bad that you can follow and do to succeed in this country Zambia.” – Community member, female, Choma district “People have praised the books especially on the folktales. Children who are born these days do not know the folktales so they thank the government that they made such an initiative to also teach children old things.” – Community member, female, Choma district

**Ethicality**	**1. Stories teach life lessons and values:** teaches children (and adults) lessons about how to live well, about love, forgiveness, humility, work ethic, respect, etc.; lessons are important to children’s growth; lessons guide children and families in the way of living well; adults also find value in the lessons; morals in line with religious beliefs	“This book teaches about how we are supposed to help each other or how to teach children on how they are supposed to help each other.” – Community member, female, Kalomo district “The stories teach that you are not supposed to [seek] revenge, like one story of the crocodile, which … shows that you are not supposed to do bad things to your friend. When someone wrongs you, you should learn to forgive.” – Community member, female, Pemba district “I am telling the story of how the lion learned to be thankful. He was helped by an antelope from a ditch but at the end he wanted to eat it. We take the lesson of being thankful to the person who has helped you not that you go against someone who is helping you.” - Community member, female, Choma district

**Self-Efficacy**	**1. Pictures support narrative:** make book easy to use: help children understand stories; support and reinforce lessons being taught **2. Book written in local language:** easier for caregivers and children to use **3. Language easy to understand:** language is simple; appropriate to read to young children	“Because in the book there are pictures that we can see, so when we are looking at those pictures, we are able to tell what the story means, even if you don’t know how to read that story, [the pictures] can guide you.” – Community member, female, Kalomo district “What makes it easy, it is not only in one language it is in all languages. If I cannot read English, then Chitonga will be easier for me to read for the child.” – Community member, female, Pemba district “[Community members] feel good about the stories, and how they are written and how they are drawn. They help to relate when you are reading, cause if you read here is a rabbit, and you will actually see the rabbit in the pictures.” – Community member, female, Choma district

**Intervention Coherence**	**1. Read at home:** caregivers and other family members read to children; set aside or prioritize time to sit together and read; parents and older siblings primarily read to young children **2. Read in groups outside the home:** groups of friends and neighbors sit together and read; older children read to younger children **3. Book facilitates dialogue:** caregivers ask children questions about the story and/or pictures; comment and explain stories and lessons to children **4. Children identify objects in pictures** **5. Pictures facilitate independent use:** children look at book independently particularly the pictures; young children sit together and look at books; children tell each other stories	“The way we use it in my family, the time we are chatting in the evenings after eating that is when we sit and start reading stories and teaching the children.” – Community member, female, Kalomo district “We do not only read for the children alone. When we read for our children, they also tell their friends to come at this time so the mother or grandmother can read for them too.” – Community member, female, Kalomo district “I sit my children down after eating and tell them to bring the book and so I ask them which story do you like the most, and so they show me and I read for them.” – Community member, female, Pemba district “When I have finished reading them a story…I will not just leave it there. I will explain… how we are supposed to live as Christians. If you have something, you have to share with your friends. That is want God requires he does not like stingy people. I tell them everything about heaven and earth.” – Community member, female, Kalomo district “As I read, I show the child things [in the pictures].” – Community member, female, Nyimba district “Children find time alone that they sit and read. Or the time they are herding goats, you find those that are able to read, they take it with them. …Those who are not yet able to read also envy it, like ‘this one knows how to read, I also want to know how to read.’” – Community member, female, Kalomo district

**Perceived Effectiveness**	**1. Children are learning and being prepared for school:** children learn vocabulary and reading comprehension; reading develops children’s brains and speech; pictures teach children to identify objects as they will in school; prepares children for school environment **2. Increases interactions with friends/family** **3. Teaches children how to interact with others:** book stories teach manners, respect, following instructions, etc; improves relationships	“The book helps to children who are supposed to start grade one because it has words a child who is starting grade one should know what they mean.” – Community member, female, Pemba district “I have seen when the children listen to these stories and when they sit with their fellow children, they share the stories saying, ‘my mother told me this and that from the book’. So that is how that friendship is going to be strong because they share new things that they didn’t know.” – Community member, female, Nyimba district “At home, if [children] are found with something and the friends do not have [the same] and the child is stingy then I just tell him that ‘You have forgotten what we were saying, what did we read in the book?’ Then you just see the child sharing with the friend.” – Community member, female, Nyimba district

**Burden**	**1. Nothing difficult:** caregivers find book easy to understand and use **2. Adult Illiteracy:** families without a family member who can read have a difficult time using the book; some vocabulary was challenging	“The only difficulty that can be there is if you are not able to read. What they read they do, they just do not know how to read then they cannot be able to know the goodness in that book.” – Community member, female, Choma district “[Some households] do not have anyone who can read, so sometimes for those who really want to know the stories, [the caregivers] will go to one who knows how to read in a group or ask that they teach them how to read. [They will say,] ‘This thing has really interested me, please teach me so that even my children should know it.’” – Community member, female, Kalomo district

**Opportunity Costs**	*No themes arose*	*No themes arose*


### Acceptability component construct: Ethicality

Respondents overwhelmingly valued the stories for their lessons explaining and encouraging positive values and morals. Respondents described the books as containing many different types of songs and stories that teach children how to live well, the virtue of patience, how to interact with others, to show respect, not to be selfish, the importance of hard work, among others. Respondents often recited to their FGD members a favorite story of theirs or their children in its entirety, describing the characters, major plot points, and the lessons taught.

### Acceptability component construct: Self-efficacy

When asked what makes the children’s book easy to use, respondents identified two main aspects of the books: (1) engaging pictures and (2) written in local language. Respondents appreciated that the pictures correlated with the story narrative, as they helped guide the reader, regardless of the reader’s age, and reinforced the lessons being taught. They explained that having pictures throughout the book makes the stories easy to understand for children as well as for less educated and/or illiterate adults who may otherwise have a hard time following each story.

Additionally, by being written in local languages, the books were easy for people to read and understand as far more individuals are fluent and literate in Chitonga and Chinyanja than in English. Several respondents specifically discussed appreciating the style of language used in the book because they find the style simple and very easy to understand without challenging words.

### Acceptability component construct: Intervention coherence

Respondents described using the books in two main ways: (1) at home, and (2) in groups of friends or neighbors. Though the respondents primarily stated, “I read,” implying that it is the female caregivers in the house who read to the young children, multiple respondents stated that “the parents read” or specifically described the children’s father reading, sometimes by bringing the wife and children together to read as a family. Respondents also described older children in the household or another family member sitting you the younger children. Some respondents described families setting time aside before bed or after school to regularly integrate reading into the household routine. Within the community, friends, or neighbors gather and read as a group or children sit together and look at the pictures or share stories they heard from the books.

Reading was often described as a more wholistic activity that also included looking at the pictures together, using the pictures to describe what is going on in the text, and discussing the lessons and values from the stories. Respondents reported children frequently identifying objects within the pictures and connecting them to the stories or to real life, while readers asked questions or provided commentary on the object names, colors, or actions. Respondents reported that the pictures enhanced the experience of reading with children by allowing them to interact in ways as independent from the written narrative. The pictures also facilitated independent use of the book by young children who looked at the many colorful scenes even when no one was available to read to them.

### Acceptability component construct: Perceived effectiveness

Respondents largely agreed that learning for children starts at home, well before they begin attending school, and is a necessary part of preparing children for grade 1. They believed that the story lessons improved children’s growth, development, and relationship skills. Respondents agreed that the book serves as an introduction to education and can help facilitate early learning. They believed the book teaches children vocabulary, helps them to develop speech and learn words earlier. The pictures teach children to identify objects as they will in school, while the pictures, reading and singing, “activate” children’s brains. Respondents believed their children are “becoming more knowledgeable,” and the books are developing their children’s’ interest in learning and school. Many respondents believed their children would be able to read earlier than others because “their brains are growing.”

Generally, respondents described that the books foster interactions within families and between children and their friends/peers. Children also learn lessons about respect, sharing, kindness, and manners from the stories, which encourages them to be better friends and family members and strengthens those relationships.

### Acceptability component construct: Burden

When asked about barriers to using the children’s book, many respondents stated that there were none. Those who did express challenges were primarily concerned with caregivers who struggle with reading and writing. A few respondents emphasized that reading the book was challenging for households without a literate adult or with adults who had received minimal formal education. Some eligible households declined the book because they did not have someone at home capable of reading it. When asked about addressing challenges with use, respondents suggested forming reading groups, in which individuals who can read would help others to use the book.

Respondents noted that caregivers without strong reading and writing skills often look to other family members, friends, and neighbors to assist in using the books by reading the books to children, or teaching the caregivers, themselves, to read.

## Discussion

Caregiver-child interactions, particularly verbal engagement with the child from birth, is critically important for child language development and emergent literacy. Communities which historically spoke non-written languages developed strong oral traditions for passing on information through generations. These traditions are still alive today and serve as a critical form of caregiver-child interactions. Shared reading experiences with young children serve as an additional platform for caregiver-child interactions and impacts children’s language, literacy, and social emotional development [6]. The presence of children’s books in the household is independently associated with child developmental outcomes [6,9].

Though not uncommon in LMICs, the impact of book distribution programs, and often the programs themselves, are not well documented. However, the need in many of these contexts is great, with the presence of children’s book in households notably sparce, which underscores the potential benefits of children’s book distribution programs in LMICs [14]. In this paper, we assessed the acceptability of the locally developed Zambian Folktales Children’s Book by the target population. Evaluation of acceptability of interventions has become more widely recognized as a critical aspect of implementation science studies and can partially explain intervention uptake, affecting resultant impacts.

Our qualitative data suggest that rural Zambian caregivers liked many aspects of the children’s book, felt that it engaged and entertained their children, believed it to be culturally appropriate for its folktale structure, and appreciated the morals and lessons the stories taught. According to our qualitative data, the Zambian Folktales Children’s Book may serve as a platform for an additional kind of interaction between caregivers and children in the home. Respondents reported using the books to read to their young children in individual or group settings, with one or both parents, an elder sibling, or other relative reading the written narratives.

Caregiver-child interaction through shared reading experiences can impact children’s emergent literacy skills and enhance their vocabulary development and listening comprehension skills [27,28,29,30]. Literature has shown that children benefit most from shared reading experiences when those experiences involve dialogue and activities outside of just reading the written narrative [27,30,31]. Reader “prompts” to encourage greater engagement by young children in the written narrative or pictures result in more verbal exchanges between reader and child and allow for additional, possibly more advanced, vocabulary use compared to what is used daily within the environment (household or educational setting) [27,30,31]. More child talk during these interactions is associated with increased vocabulary development [31]. Respondents in our study of the children’s book reported a wide variety of other ways in which the book was fostering interactions outside of just reading the written narrative. Respondents prompted children to identify objects or answer questions and they described aspects of the pictures to the children, such as colors, animals, and actions. Respondents frequently discussed children looking at the pictures independently when not being read to.

Increases in caregiver-child interactions around shared reading activities have been shown in assessments of other book distribution programs [12,32]. Increases in caregiver-child interactions may affect parenting methods in these households more widely, and have been shown in other book distribution studies to impact child development outcomes [6,12]. In particular, the books may be fostering specifically father-child interactions, though this was only discussed briefly by respondents. Father involvement and interactions with children in early childhood is an important factor in the cognitive and social emotional development of young children [33]. Furthermore, the books may foster other interactions with non-primary caregiving adults and young children, providing additional and different opportunities for children to develop and practice their social emotional skills.

Conclusions on the impact of this children’s book should not be drawn from our qualitative data alone. Children’s book interventions with caregivers which have shown impacts on child developmental outcomes have often been accompanied by health messaging or workshops with caregivers to educate them on the importance of reading with young children and practice this skill [6,10,12]. Evidence on the efficacy of interventions that only distribute books, without broader messaging or parental workshops is sparse. Additionally, the style and quality of engagement and non-narrative discourse between reader and child during the shared reading experience has been shown to vary substantially [27,29,31,34,35], affecting the potential benefits to the child [27,29,30,31,35,36]. This highlights the importance of providing additional interventions with the primary readers to ensure the children are receiving the greatest possible benefits. Though the overarching evaluation which included this study also included twice monthly parenting groups with caregivers of children under five that emphasized the importance of reading to children [25], many of the recipients of the Zambian Folktales Children’s Book did not participate in these parenting groups and, therefore, may not have received the full benefits of the combined intervention, including understanding how to use the book to its fullest potential with their young children. An assessment of the types and quality of shared reading experiences with the children’s book as well as the impacts of the book on child developmental outcomes would be beneficial among households participating and not participating in parent educations groups to add to the literature.

With the low levels of literacy in the rural Zambian adult population – only 54% and 74% of rural Zambian women and men, respectively, are considered literate [18] – while still utilizing the book to foster caregiver-child interactions around pictures, some households may specifically struggle to utilize the children’s book as a tool to foster early reading. However, those caregivers may also substantially benefit from the introduction of a simple and engaging book if they participate in reading circles where literate older children, relatives, or neighbors read the stories aloud, as suggested in our findings. Caregivers in this study found the book to be well written with simple vocabulary in the local languages and engaging pictures that enhance the reading experience, facilitating the use of the book even by less educated caregivers. These community reading circles may have additional benefits to young children, as Dowd et al. (2017) has shown among study participants in Asia and Africa [8]. Future book distribution programs may want to consider messaging encouraging both at-home reading with caregivers as well as community reading circles particularly in low-literacy settings.

Households with caregivers who have low skills in reading and writing may still have strong oral literacy skills and may be just as effective in utilizing the book to interact with young children and foster their language and social emotional development without focusing on reading. The introduction within the book indicates its intention to foster dialogue around the pictures and to stimulate the telling of additional folktales stories not included in the book, a historically and culturally important tradition among these communities [23]. Though not discussed by respondents, this would be an important means of fostering caregiver-child interactions among caregivers who have low skills in reading and writing.

### Limitations

While one of the first of its kind to assess the community acceptability of a freely distributed, locally designed children’s book in low resource settings, this study does have several limitations. Since this was a qualitative study, we could not assess the real-world use or impact of the children’s book. We can only report on the perceptions of female caregivers of young children. An assessment of at home book use and language, early literacy, and listening comprehension assessments to determine the potential impact of this early reading intervention are needed. Additionally, this study does not report on the cost or cost effectiveness of this intervention. It would be beneficial to understand the costs associated with developing and distributing the children’s book, especially compared to any positive outcomes that may be found in children’s development.

Furthermore, only the perspectives of female caregivers are included in this study. Though these are the primary caregivers in the rural Zambian context who interact with children the most, it would be beneficial to understand the perspectives of male caregivers and community elders who may or may not utilize the children’s books and read to young children.

In addition, respondents were not asked about specific kinds of barriers, such as limited time to read with children or opportunity costs to using the children’s book. This would have provided valuable information on challenges and the potential long-term success of the intervention.

Lastly, as these were FGDs, we likely missed hearing from the caregivers who do not have strong skills in reading and writing or we, at least, are not able to ascertain who they might be in the data. Future work should seek to understand more from these caregivers, their perceptions and use of the book, as they likely engage with the book in different but equally important ways.

## Conclusion

Though the presence of age-appropriate books in households has been widely associated with multiple aspects of children’s development including language development and emergent literacy skills, little is known if the millions of books distributed annually to vulnerable households are impacting these skills and improving the developmental prospects of children facing the greatest adversity. More external research, and publication of program descriptions and internal evaluations, is needed to determine the true impact of these promising interventions. With limited literature on similar interventions in low resource settings, this study offers valuable information for future funders and implementers looking to replicate this experience. The results of this qualitative study suggest that a locally developed children’s books designed to be age appropriate and culturally relevant for a rural population could be well received and utilized even in low literacy settings in sub-Saharan Africa facing substantial adversity. While the generally positive perceptions of this book are encouraging, and we recommend empirically evaluating the children’s book for impact on child language development and emergent literacy.

## Reflexivity statement

Here we provide details on the authors’ nationalities, seniority, fields of expertise, and experiences in the cultures and communities involved in this study. TN and BC are Zambian; GF is Swiss, and the remaining authors are American. BC and DHH are medical doctors. NAS, GF, and PCR have doctorates in public health and economics, respectively. JLK, TN, and AJ have master’s degrees in public health. NAS, JLK, TN, and AJ are primarily program evaluators with expertise in mixed-methods program and impact evaluations. GF and PCR have expertise in ECD in Zambia and sub-Saharan Africa more broadly, developing contextually appropriate ECD assessment tools, and conducting ECD research studies and program evaluations to better understand current influencers of ECD in these context and methods to help children achieve their full potential. DHH is an expert in infectious diseases and in maternal and child health and nutrition, while BC has extensive experience in HIV/AIDS. The American and Swiss authors and TN have been working with the communities of Southern and Eastern Provinces to improve maternal and child health and developmental outcomes for 15 years on average per person. JLK, NAS, and DHH have each lived in Zambia for a combined period of 15 years, much of that time spent in rural towns in Southern and Eastern Provinces learning from and about their cultures and communities.

While all authors have made notable efforts to engage with and learn from the rural Zambian communities we work with, we recognize there is always more to learn and, for those of us who are cultural outsiders, a need to remove our cultural assumptions to the degree possible. We believe one of the key roles of qualitative studies is to allow communities to speak for themselves, with many direct quotes presented throughout the manuscript.

Though many of us had previously worked with the primary developer of the Zambian Folktales Children’s Book, as external evaluators personally uninvolved in the development of the book, we made no assumptions regarding the community’s perceptions of the book and their use of it. While some authors (TN and BC) were involved in widespread distribution of the books under a grant from USAID, they were equally interested in understanding the true community perceptions of the books and its potential future usefulness.

## Data Accessibility Statement

Data from these FGDs will be made available upon acceptance of this article in a public, open access repository at https://hdl.handle.net/2144/46056.

## Additional File

The additional file for this article can be found as follows:

10.5334/aogh.3920.s1 Supporting File 1. Focus Group Discussion Guide with Caregivers.

## Author Information

The authors report their institutions received grants directly or indirectly from the United States Agency for International Development (USAID) Zambia to conduct the work.
