# Supplementary material for: A Qualitative Assessment of Community Acceptability and Use of a Locally Developed Children’s Book to Increase Shared Reading and Parent-Child Interactions in Rural Zambia
Source: Ann Glob Health. 2023 Apr 27;89(1):28. doi: 10.5334/aogh.3920 (PMC10143943; doi:10.5334/aogh.3920)
Supplement: Supporting File 1. — Focus Group Discussion Guide with Caregivers. [file agh-89-1-3920-s1.pdf]

GROUP ID [ ]

Instrument ID

The SUpErCDZ Project

Process Evaluation – **Focus Group Discussion Guide with Caregivers**
ENGLISH

Target Audience:

1. Women with child 3-9 years old, >=18 years at last birthday

6-8 participants per FGD

Short screen:

**Criteria**	**Response**	**Comments**
SS1. Does the caregiver have a child 3-9 years old?	Yes (1)<br>No (0)	If (0), do not proceed. Person is not eligible.
SS2. Is the caregiver >=18 years old?	Yes (1)<br>No (0)	If (0), do not proceed. Person is not eligible.
SS3. Has the caregiver previously participated in a focus group in the same round at the same or another site or other categories?	Yes (1)<br>No (0)	If (1), do not proceed. Person is not eligible.

Instructions for the interviewer

Step 1: Informed consent: Ask each participant for a few minutes of their time. Introduce yourself and the study. Begin the informed consent process as per the training.

Was informed consent obtained and documented for each participant before the start of the FGD?

YES ______ (proceed with roster and FGD)

NO ______ (STOP! Thank the participant for their time but ask any who did not agree to leave. Then proceed with the FGD.)

Interviewer*: Read the following statement. Please repeat the statement translated into the local language based on primary languages used by the group.*

"Thank you for agreeing to participate in this interview. My name is ____________________. I will be asking you the questions. My partner ___________________ will be taking notes on the things you have to say. We will record this session.

We want to understand your and your community's views on the Zambian Folk Tales Children's Book intervention. We would also like to understand your activities in COVID-19 information sharing and Ministry of Health guideline enforcement. Please feel free to tell us whatever you are comfortable sharing. As a

reminder, please do not share anything you hear or said within this group outside of this group. You should also remember that you do not have to share anything you are not comfortable sharing. We will not link your information to your responses. There are no right or wrong answers, so please be honest and us what is true for you and your community. Are you ready to begin?”

Step 2: *Please complete the roster table for each participant as they sign in. Verify eligibility. This form will have an ID letter for each participant. Make sure that the note taker has the correct ID letter recorded on his/her notes prior to beginning and that participants have their correct “Letter Label”.*

Step 3: *Proceed to the FGD. Start by reiterating the importance of confidentiality within the group. As you ask the questions, please probe to obtain as much information as possible for each question.*

Facilitator name ________________________ **Note-taker Name** _____________________

1. **FGD Date** | | | | | | | | | (DD MM YYYY)

2. **Health Facility Name** ________________________

3. **District Name** ________________________

4. **Province Name** ________________________

5. **Time Start:** [H H] : [M M]

6. **Time Finish:** [H H] : [M M]

GROUP ID

Demographics

ID	Did participate in another FGD this round?<br>*No (0)*<br>*Yes (1)*<br>*If Yes (1), person not eligible to participate*	Age at last Birthday<br>*If <18 years old, person not eligible to participate*	Are you the caregiver of any child 3-9 years of age?<br>*Yes (1)*<br>*No (0)*<br>*If No (0), person not eligible to participate*	Are you a resident of [*name of zone*]?<br>*No (0)*<br>*Yes (1)*	Eligibility confirmed?<br>*Yes (1)*<br>*No (0)*<br>*If 1, proceed with the rest of the columns*<br>*If 0, stop and thank the woman for her time*	Zone name<br>*(where caregiver lives)*	Village name<br>(where caregiver lives)	Gender<br>*Male (1)*<br>*Female (2)*	Marital status:<br>*Married/ cohabiting (1)*<br>*Divorced (2)*<br>*Separated (3)*<br>*Widowed (4)*<br>*Never married (5)*	What is the highest grade you completed?<br>*If < Grade 1, write 0.*<br>*If > Grade 12, write 13.*	How many children <5 live in your household?	How many children 5-9 live in your household?	How many children 10-17 live in your household?	Has your household received the Zambian Folk Tales Children's Book?<br>*No (0)*<br>*Yes (1)*
**A**														
**B**														
**C**														
**D**														

GROUP ID

ID	Did participate in another FGD this round?<br>*No (0)*<br>*Yes (1)*<br>*If Yes (1), person not eligible to participate*	Age at last Birthday<br>*If <18 years old, person not eligible to participate*	Are you the caregiver of any child 3-9 years of age?<br>*Yes (1)*<br>*No (0)*<br>*If No (0), person not eligible to participate*	Are you a resident of [*name of zone*]?<br>*No (0)*<br>*Yes (1)*	Eligibility confirmed?<br>*Yes (1)*<br>*No (0)*<br>*If 1, proceed with the rest of the columns*<br>*If 0, stop and thank the woman for her time*	Zone name<br>*(where caregiver lives)*	Village name<br>(where caregiver lives)	Gender<br>*Male (1)*<br>*Female (2)*	Marital status:<br>*Married/ cohabiting (1)*<br>*Divorced (2)*<br>*Separated (3)*<br>*Widowed (4)*<br>*Never married (5)*	What is the highest grade you completed?<br>*If < Grade 1, write 0.*<br>*If > Grade 12, write 13.*	How many children <5 live in your household?	How many children 5-9 live in your household?	How many children 10-17 live in your household?	Has your household received the Zambian Folk Tales Children's Book?<br>*No (0)*<br>*Yes (1)*
**E**														
**F**														
**G**														
**H**														

GROUP ID

<u>Theme 1: Zambian Folk Tales Children's Book</u>

"In my first set of questions, I am going to ask you about the preparing a child for Grade 1 and the Zambian Folk Tales Children's Book."

1a. What do you think a **child needs in order to be ready for Grade 1**?

Follow up questions

i. What does a child need to know?

ii. What does a child need to be able to do?

iii. How does a child learn these things?

iv. What is the role of the caregiver in preparing a child for Grade 1?

1b. In general, what **types of stories** do people in this community tell their children, if any? Please give me an example.

After respondent answers, probe for:

- Family stories
- Folklore stories: Reading or telling from memory?
- Religious stories: Reading or telling from memory?
- Other: Reading or telling from memory?
- None

1c. At **what age** do people in this community start telling children stories?

1d. Can you tell me **what you know about the Zambian Folk Tales Children's Book,** if anything?

Follow-up questions

i. What kinds of stories does it contain?

ii. What age group does it target?

1e. How was the **book distributed**? Please tell me about general experiences in your community.

Follow-up question

i. Do you think distribution was successful? Why or why not?

ii. What kinds of households received the book?

i. Do you think all households with a child 9 years of age and under were given a book? Why or why not?

1f. What have you **heard from the community** about the Zambian Folklore Children's Book?

Follow-up questions

i. What does the community think about the stories included in the book?

ii. What does the community think about the pictures in the book?

iii. What does the community think about the questions included at the end of each story?

1g. What do people say is **easy about using the book**? Please give examples.

**** **(Facilitator: MAKE SURE TO PROBE!)** ****

1h. What do people say is **challenging about using the book**? Please give examples.

**** **(Facilitator: MAKE SURE TO PROBE!)** ****

Follow-up question

i. What could be done to address those challenges?

1i. How is the **book being used** within the community?

i. How is it used within parenting groups (if applicable)?

ii. How is it used within the home?

iii. Who is reading to the children?

iv. How are caregivers who are unable to read using the book, it at all?

v. If a caregiver is unable to read, who reads to the children?

vi. Are there other ways the book is being used within the community?

1j. Do you think the book affects **caregiver-child and child-child interactions** within households?

1k. Do you think the book helps **prepare children for school**? Why or why not?

1l. What **types of children's books** do people in your community want?

Follow-up question

i. How does the Zambian Folk Tales Children's Book compare to others books targeted at the same age range?

1m. What else is **needed to better prepare young children for Grade 1**?

[other themes removed from instrument]

"This is the end of the Focus Group. Thank you for your time."
